# Combined growth hormone and insulin-like growth factor-1 rescues growth retardation in glucocorticoid-treated *mdx*mice but does not prevent osteopenia

**DOI:** 10.1530/JOE-21-0388

**Published:** 2022-02-21

**Authors:** Claire L Wood, Rob van ‘t Hof, Scott Dillon, Volker Straub, Sze C Wong, S Faisal Ahmed, Colin Farquharson

**Affiliations:** 1 Division of Functional Genetics and Development, Roslin Institute, University of Edinburgh, Edinburgh, UK; 2 Translational and Clinical Research Institute, Newcastle University, Newcastle upon Tyne, UK; 3 Institute of Ageing and Chronic Disease, University of Liverpool, Liverpool, UK; 4 John Walton Muscular Dystrophy Research Centre, Newcastle University and Newcastle Hospitals NHS Foundation Trust, Newcastle upon Tyne, UK; 5 Developmental Endocrinology Research Group, School of Medicine, University of Glasgow, Glasgow, UK

**Keywords:** bone QCT/micro CT, genetic animal models, GH/IGF-1, bone–muscle interactions

## Abstract

Short stature and osteoporosis are common in Duchenne muscular dystrophy (DMD) and its pathophysiology may include an abnormality of the growth hormone/insulin-like growth factor-1 (GH/IGF-1) axis, which is further exacerbated by long-term glucocorticoid (GC) treatment. Hence, an agent that has anabolic properties and may improve linear growth would be beneficial in this setting and therefore requires further exploration. A 5-week-old x-linked muscular dystrophy (*mdx*) mice were used as a model of DMD. They were treated with prednisolone ± GH + IGF-1 for 4 weeks and then compared to control *mdx* mice to allow the study of both growth and skeletal structure. GC reduced cortical bone area, bone fraction, tissue area and volume and cortical bone volume, as assessed by micro computed tomography (CT) In addition, GC caused somatic and skeletal growth retardation but improved grip strength. The addition of GH + IGF-1 therapy rescued the somatic growth retardation and induced additional improvements in grip strength (16.9% increase, *P*  < 0.05 compared to control). There was no improvement in bone microarchitecture (assessed by micro-CT and static histomorphometry) or biomechanical properties (assessed by three-point bending). Serum bone turnover markers (Serum procollagen 1 intact N-terminal propeptide (P1NP), alpha C-terminal telopeptide (αCTX)) also remained unaffected. Further work is needed to maximise these gains before proceeding to clinical trials in boys with DMD.

## Introduction

Duchenne muscular dystrophy (DMD) affects 1 in 4000 live male births and is a severe and ultimately fatal disease (Crisafulli *et al.* 2020) caused by mutations in the *DMD*gene at 21p on the X chromosome. The reduction or loss of dystrophin protein weakens the sarcolemmal membrane and results in progressive replacement of muscle fibres by fat and fibrous tissue (Deconinck & Dan 2007). Glucocorticoids (GC) are currently the mainstay of treatment in DMD and are an established pharmacological intervention proven to stabilise muscle strength for a finite period of time (Manzur *et al.* 2008, Moxley *et al.* 2010, Birnkrant *et al.* 2018). The side-effects of GC, however, are well recognised, with growth retardation and bone fragility being extremely common. To date, there remains a limited effective therapy for the management of poor growth and osteoporosis in these boys. Linear bone growth is known to be a driver for improvements in bone health in children. In addition, impaired osteoblast function has been described in both X-linked muscular dystrophy (*mdx*) mice and patients with DMD (Rufo *et al.* 2011). For that reason, an agent that has anabolic properties and may improve linear growth would be beneficial in this setting and therefore requires further exploration.

Growth hormone (GH) and insulin-like growth factor (IGF-1) are fundamental regulators of longitudinal bone growth and have synergistic effects as well as independent roles in the regulation of growth, bone and muscle development (Lupu *et al.* 2001, Giustina *et al.* 2008, Cohen *et al.* 2014, Ahmad *et al.* 2020). GC-treated boys with DMD show GH insufficiency with normal IGF-1 levels (Rutter *et al.* 2012); long-term GC treatment in people with chronic diseases may also induce functional GH resistance (Mushtaq & Ahmed 2002, Wong *et al.* 2016). The use of recombinant human (rh) GH or rhIGF-1 has been shown to increase short-term linear growth in preliminary studies in boys with DMD, but as neither study included any bone outcome measures, further study is required (Rutter *et al.* 2012, 2020). Furthermore, co-administration of GH and IGF-1 may lead to more favourable outcomes than administered individually (Backeljauw *et al.* 2015). Given that both rhGH and rhIGF-1 are licensed for use in children with growth disorders and in light of data from children and adults with chronic inflammation, there is potential for these anabolic agents to improve growth, muscle strength and bone mass, which are fundamental problems in patients with DMD (Bucuvalas *et al.* 2001, Mauras *et al.* 2002). Before trialling these agents in combination, an improved understanding of (1) their effects on linear growth and bone mass, and (2) the underlying mechanisms through which they exert their effects on bone, is imperative. The *mdx* mouse is the most commonly used and best characterised animal model of DMD. Although it has limitations as its phenotype is less severe than in DMD (Wood *et al.* 2020), juvenile GC-treated *mdx* mice display growth retardation and cortical bone defects (Yoon *et al.* 2016, 2019*a*).

This study aimed to determine whether the combined administration of rhGH and IGF-1 could rescue the GC-induced skeletal impairment and growth retardation in juvenile *mdx* mice.

## Materials and methods

### Animals and experimental procedures

*Mdx*mice (C57BL/10ScSn.mdx) were obtained from Jackson laboratories. In the UK, a Project License is required for any regulated animal work. The Animal Welfare and Ethical Review Body (AWERB) reviewed Project License PFB9E8295, and the license was granted by the Home Office on the 10th of October 2019. Additionally, before a study begins, an individual study plan is written and a member of the AWERB, the named veterinary surgeon reviews this document for implementation of the 3Rs and compliance. This study was conducted in line with the Home Office Code of practice (for the housing and care of animals bred, supplied or used for scientific purposes). All mice were housed under controlled temperature (approx. 25°C) and light conditions (12 h light:12 h darkness cycle) and offered food and water *ad libitum*. Six to eight male mice were used in each interventional group. Bodyweight (BW), crown-to-rump and tail length measurements were taken twice weekly from day 32 until cull. Testes were dissected immediately post cull and weighed using calibrated weighing scales. Combined weights are presented, alongside weight normalised to BW.

Rapidly growing 5-week-old male *mdx* mice were treated with prednisolone ± GH + IGF-1 for 4 weeks to allow the study of both growth and skeletal structure. A 4-week treatment with prednisolone has previously been shown to result in growth retardation and poor bone health (Yao *et al.* 2016). The three different experimental groups were: (1) control group: mdx mice given cherry syrup vehicle by daily oral gavage, twice daily s.d. injection of saline vehicle and saline vehicle (via osmotic pump), (2) prednisolone (pred) group: *mdx* mice given pred (20 mg/kg/day by oral gavage chosen after dose-finding studies; incremental regimens of 5, 10 and then 20 mg/kg were used after extensive literature review (Sali *et al.* 2012, Wood *et al.* 2018) (data not shown)) in cherry syrup, twice daily s.d. injection of saline vehicle and saline vehicle (via osmotic pump) and (3) GH + IGF-1 treated group: *mdx* mice given a combination of pred (20 mg/kg/day by oral gavage) in cherry syrup, rhGH (3 mg/kg via twice daily s.d. injection) (Masternak *et al.* 2010, Dobie *et al.* 2014) and IGF-1 (1 mg/kg/day via osmotic pump) (Gregorevic *et al.* 2002, Schertzer *et al.* 2006). The decision to use combination therapy was based on previous studies that had not demonstrated an effect of exogenous GH on either growth in male mice (Dobie *et al.* 2015) or long bone microarchitecture/bone density in GC and GH-treated *mdx* mice (Yoon *et al.* 2019*a*). No clinical studies have assessed a combination of GH and IGF-1 therapy, but separate studies have shown improved linear growth; a doubling of height velocity, and an increase in lean muscle mass were demonstrated after 6 months of IGF-1 treatment in boys with DMD (Rutter *et al.* 2020). Height velocity also increased in an open-label observational study where 39 boys with DMD were treated with GH for a year, but the rate of weight gain was unchanged (Rutter *et al.* 2012).

### Administration of GH and IGF-1 or vehicle

rhGH in the form of norditropin (Novo Nordisk) was diluted using sterile 0.9% saline and 3 mg/kg was administered twice daily by s.d. injection, except at weekends when only one dose was given daily due to staffing constraints. Biotinylated IGF-1 (Gro-Pep, Adelaide, Australia) or the equivalent volume of physiological saline was given by micro-osmotic pump (Alzet model 1004, California, USA). The pump was implanted behind the scapula, on day 32. Forty-eight hours was given for wound healing before the s.d. injections and oral gavages were commenced. No post-intervention complications were noted, and all mice were able to progress into the main study period.

### Grip strength

Forelimb grip strength testing was performed within 24 h prior to cull, using a grip strength metre with a specialised mouse grid (Harvard Biosciences, Massachusetts, USA), according to the TREAT-NMD standard operating protocol (De Luca 2014). All testing was carried out between 8:00 and 09:00 h by the same animal technician who was blinded to the treatment group. The mean of three consecutive measurements was calculated and normalised to BW.

### Serum measurements

Mice were culled by exsanguination (non-schedule one method), under terminal anaesthesia with confirmation by cervical dislocation. Immediately following sacrification, blood was obtained from non-fasted animals via cardiac puncture. Serum was extracted and stored at −80°C until required. Serum creatine kinase (CK) activity was measured by a CK assay kit (Pointe Scientific, Stroud, UK). The change in NAD phosphate (NADPH) absorbance was measured every 30 s at 340 nm for 4 min at 25°C and the mean value was calculated. Serum procollagen 1 intact N-terminal propeptide (P1NP) and alpha C-terminal telopeptide (αCTX) concentrations were measured by ELISA (AMS Biotechnology, Abingdon, UK). Each serum sample was tested in duplicate.

### Micro-CT analysis

Micro-computed tomography imaging (μCT) was performed to quantify trabecular architecture, cortical bone geometry, tissue mineral density (TMD) and tibia length. Left tibiae were dissected from all mice, stored in H_2_O at −20°C and thawed prior to scanning with a SkyScan 1272 X-ray microtomograph (Bruker Corporation, Kontich, Belgium) as previously described (Wood *et al.* 2020). For trabecular bone, images were obtained at a 4.5 µm resolution, with the source at 50 kV, using a 0.5 mm aluminium filter and 0.3° rotation step. For cortical bone image accrual and tibial length measurements, images were obtained at a 9 µm resolution using a 0.5 mm aluminium filter with a 0.5° rotation step. Scans were reconstructed using NRecon software (Bruker), and a volume of interest was selected using Data Viewer software (Bruker). Two hundred slices of the metaphysis were taken for the analysis of trabecular bone, with the region of interest (ROI) starting ten slices below the base of the growth plate (GP). One hundred slices of the diaphysis were taken for the analysis of cortical bone, with the ROI starting 50 slices above the tibia–fibula junction. CTAn software (Bruker) was used to analyse appropriate parameters. Cortical TMD was calculated after calibration using a pair of hydroxyapatite rod phantoms of a known density (0.25 g/cm^3^ and 0.75 g/cm^3^), which were scanned using the same settings used for cortical bone image acquisition.

### Biomechanical testing

Three-point bending analysis of the cortical region of the left tibiae was carried out immediately after μCT, using a Lloyd LRX5 materials testing machine (Lloyd Instruments, West Sussex, UK). A 100 N loading cell was used with the span fixed at 10 mm and the cross-head was lowered at 1 mm/min to determine the load to failure and maximum stiffness and deflection of tibiae (Huesa *et al.* 2011).

### Bone histomorphometry

Right tibiae were fixed in 10% neutral buffered formalin for 24 h and then decalcified for 3 weeks in 10% ethylenediaminetetraacetic acid and processed to wax using standard procedures. Sections were stained with toluidine blue to enable the calculation of total and zonal widths of proliferating and hypertrophic zones of the GP (Owen *et al.* 2009). Ten measurements were taken per section and the mean height was calculated for each zone. Static histomorphometry was performed on paraffin-embedded, decalcified sections of proximal tibiae. Tartrate-resistant acid phosphatase (TRAP) staining was used to identify osteoclasts (TRAP+ve) as multinucleated cells lying on the bone surface. Slides were stained with Goldner’s trichrome using standard protocols to identify osteoblasts. Images were scanned using a Nanozoomer slide scanner (Hamamatsu Photonics, Iwata City, Japan), and then, the trabecular area of the proximal tibia was analysed. The ROI included only metaphyseal trabecular bone and extended from 50 µm below the GP and within the endocortical bone boundary. Osteoblast and osteoclast numbers per bone surface were determined using BioqantOsteo v 11.2.6 (Bioquant Image Analysis Corp, Nashville, Tennessee, USA), in accordance with the American Society of Bone and Mineral Research Guidelines for nomenclature (Dempster *et al.* 2013).

### Assessment of chondrocyte proliferation rate

Proliferating cell nuclear antigen (PCNA) immunohistochemistry (IHC) was performed using a 1:4000 dilution of an anti-PCNA antibody (Abcam) followed by the Vectastain elite ABC rabbit kit (Vector Labs, Peterborough, UK) on paraffin-embedded sections of proximal right tibiae. Sections were counterstained by haematoxylin and viewed using a Zeiss AxioImager brightfield microscope. The haematoxylin–DAB colour deconvolution plugin was used within Fiji to calculate the percentage of PCNA +ve cells in the proliferating zone of the GP of each sample (Schindelin *et al.* 2012).

### Muscle histology

The tibialis anterior muscles were removed and fixed in 4% paraformaldehyde before undergoing paraffin wax-embedding. Sections (6 µm) were stained with haematoxylin and eosin for histological assessment, according to the TREAT-NMD protocol (Grounds 2014*a*). Images were acquired using a Zeiss AxioImager brightfield microscope and analysed using Fiji (Schindelin *et al.* 2012). The percentage of inflammatory cells (characterised by infiltrated dystrophic myofibres with barely visible sarcoplasm) in the ROI was calculated, and the number of central nuclei (signifying muscle regeneration) was recorded, to obtain a cumulative measure of skeletal muscle damage (Grounds 2014*a*).

### Statistical analysis

Mice were identified only by number at the time of culling, to enable blinding by treatment protocol. Statistical comparisons were performed between mice of each interventional group, using STATA v15 and GraphPad Prism v7. After checking for normality of data, one-way ANOVA was used to assess the significance of differences between groups, and post-test Bonferroni modifications were made to adjust for multiple comparisons. Data are presented as mean ± s.d. A *P* -value of <0.05 was accepted as significant. μCT data were also standardised for tibial length and BW at cull, but significant results remained the same, and therefore, only unadjusted data are shown.

## Results

### Growth

**Figure 1:**
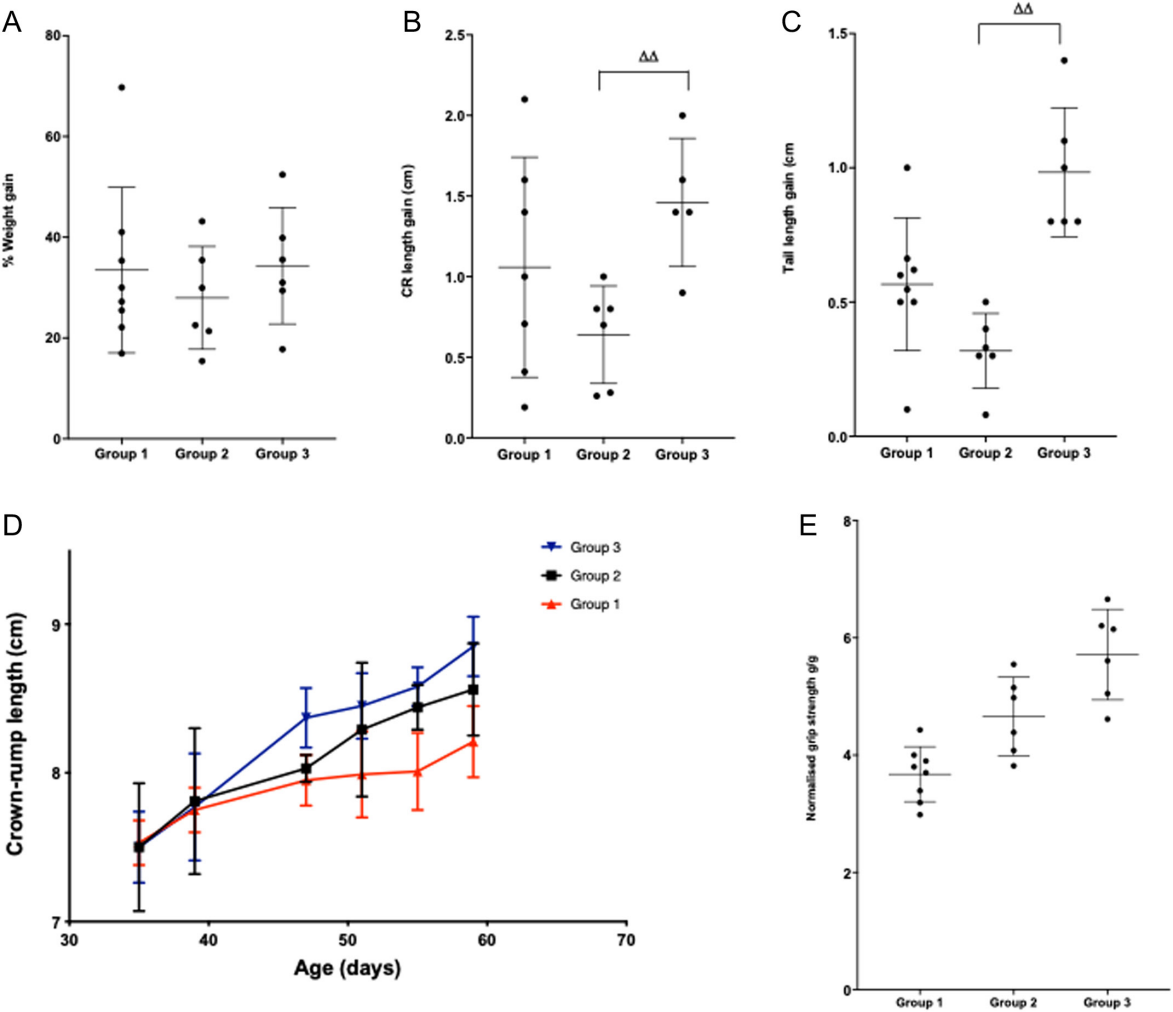
(A) Change in % weight gain during the study period, by intervention group. (B) Increase in crown-rump length during the study period, by intervention group. (C) Increase in tail length during the study period, by intervention group. (D) Mean crown-rump length at each time point during the intervention period showing the growth retardation in the *mdx* mice given prednisolone and the increased growth velocity in the *mdx* mice given rhGH and rhIGF-1 in addition to prednisolone. (E) Significant increase in normalised mean grip strength in groups 2 and 3,. *n*  = 8 in group 1 and 6 each in groups 2 and 3. Data are presented as mean (±s.d.), *P  < 0.05, **P  < 0.01, compared to group 1 (vehicle only) ^ΔΔ^*P*  < 0.01 compared to group 2 (pred + vehicle). *n*  = 8 in group 1 and 6 each in groups 2 and 3. A full colour version of this figure is available at https://doi.org/10.1530/JOE-21-0388.

BW at cull was significantly lower in pred-treated mice compared to GC-naïve controls, with the same trend also noted for a reduction in % BW gain (Fig. 1A and Table 1). The administration of rhGH and IGF-1 did not overcome the GC-induced weight deficit. In contrast to the BW data, the administration of GH and IGF-1 was sufficient to rescue both crown-to-rump length and tail length growth retardation caused by GC; mean crown-to-rump length gain of 1.35 ± 0.45 cm (GH + IGF-1 treated) vs 0.64 ± 0.30 cm (pred) and tail length gain of 0.95 ± 0.40 cm (GH + IGF-1 treated) vs 0.32 ± 0.14 cm (pred) (both *P*  < 0.01; Fig. 1 and Table 1). There were no significant differences in either testes weight or testes weight normalised to BW (Table 1). Combination GH + IGF-1 therapy was not able to rescue the reduction in tibial length caused by pred (Table 1).

**Table 1:** Change in parameters during the study period, by intervention group.

	Control	Prednisolone	Prednisolone, rhGH and IGF-1
Growth parameters			
BW at cull, g	27.35 (2.42)	21.07 (1.27)^c^	22.56 (1.71)^b^
Gain in BW, %	32.12 (17.20)	27.98 (10.21)	31.90 (13.75)
Gain in crown-rump length, cm	1.00 (0.65)	0.64 (0.30)	1.35 (0.45)^e^
Gain in tail length, cm	0.60 (0.27)	0.32 (0.14)	0.95 (0.40)^e^
Combined testes weight, g	0.12 (0.27)	0.11 (0.14)	0.14 (0.40)
Combined testes weight, normalised to BW	0.004 (0.002)	0.005 (0.002)	0.006 (0.001)
Tibial length on µCT, mm	16.91 (0.44)	15.98 (0.36)^b^	15.87 (0.49)^b^
Height of total growth plate, µm	139.39 (9.34)	139.7 (1.72)	155.96 (16.78)
Height of hypertrophic zone, µm	52.00 (6.71)	55.3 (2.34)	60.52 (7.03)
Height of proliferative zone, µm	75.03 (12.08)	68.55 (7.57)	60.52 (7.05)
PCNA +ve nuclei, %	37.4 (6.1)	36.2 (0.6)	32.2 (2.3)
Grip strength			
Absolute grip strength, g	103.13 (12.66)	98.46 (17.09)	125.41 (11.60)^b,d^
Grip strength normalised to BW	3.80 (0.61)	4.66 (0.67)a	5.61 (0.83)^b^
Serum measurements			
CK, U/L	697.70 (546.90)	916.9 (420.20)	1100.6 (243.50)
P1NP, pg/mL	59.4 (25.80)	32.5 (21.90)	47.7 (23.40)
αCTX, pg/mL	159.2 (92.0)	224.5 (61.9)	212.9 (81.4)
Histology of tibialis anterior			
Inflammatory cells, signifying active cell damage, %	2.85 (1.78)	4.35 (2.88)	2.40 (1.88)
Central nuclei, signifying regeneration, %	2.51 (1.65)	2.51 (1.09)	2.73 (1.50)
Cumulative damage, %	5.37 (2.85)	6.86 (2.86)	5.13 (3.16)
Biomechanical properties			
Maximum load, *n*	9.06 (2.38)	7.16 (1.84)	6.15 (1.16)
Deflection at max load, mm	0.65 (0.14)	0.64 (0.17)	0.62 (0.11)
Stiffness, Nm	27423 (7399)	31407 (20054)	17724 (5373)

Data are presented as mean (± s.d.),^a^denotes *P*  < 0.05, ^b^denotes *P*  < 0.01, ^c^denotes *P*  < 0.001 compared to group 1, ^d^denotes *P*  < 0.05 compared to group 2, ^e^denotes *P*  < 0.01 compared to group 2. *n*  = 8 in group 1 and 6 each in groups 2 and 3.

BW, bodyweight.

### Muscle

Absolute grip strength was higher in the GH + IGF-1 treated mice compared with pred (125.4 g vs 98.5 g, *P*  < 0.01) and control (125.4 g vs 103.1 g, *P*  < 0.05) mice. Grip strength normalised to BW was significantly greater in both the pred (4.7 vs 3.8, *P*  < 0.01) and GH + IGF-1 (5.6 vs 3.8, 16.9% increase, *P*  < 0.05) mice compared with control mice (Fig. 1 and Table 1). There was no significant change in serum CK levels by intervention group (Table 1). Histological analysis of the TA muscle in all three groups of mice revealed clear evidence of muscle necrosis with inflammatory infiltration alongside evidence of regeneration with larger, irregular muscle fibres containing central nuclei. There were no significant differences in the amount of inflammation or muscle regeneration by interventional group (Table 1).

### Bone

**Figure 2:**
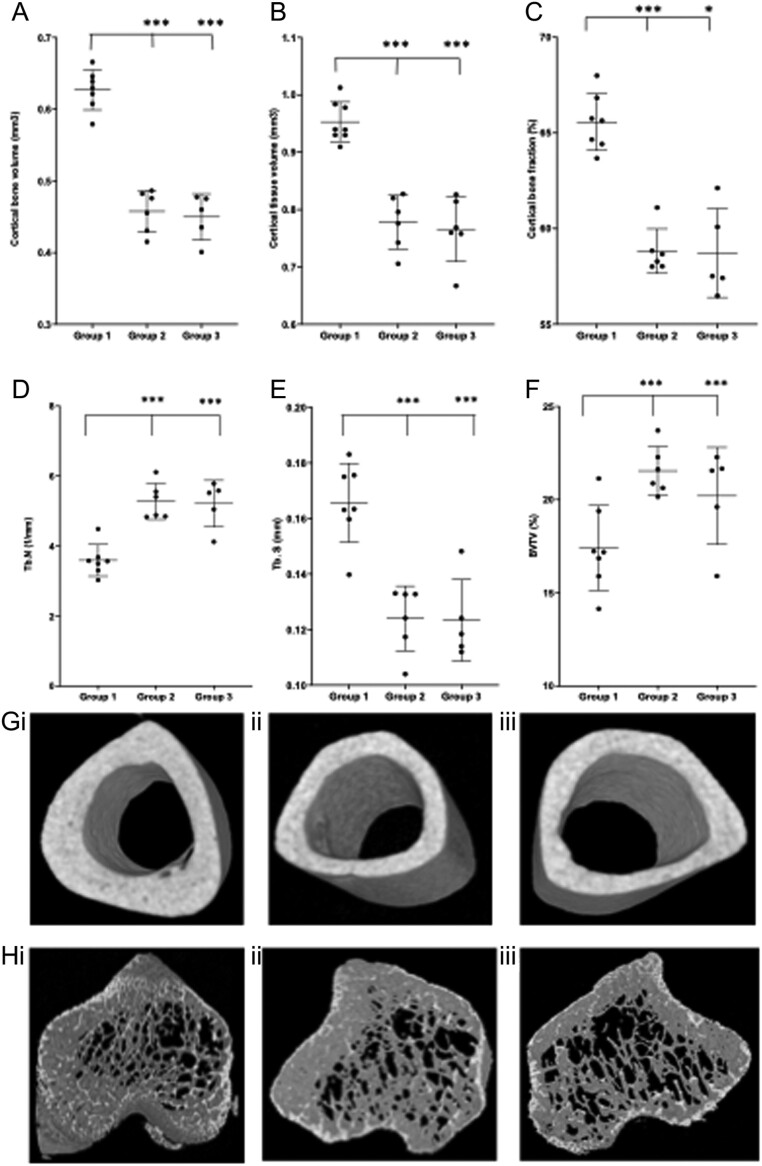
Trabecular and cortical bone parameters assessed by μCT. Graphs show reduced (A) cortical bone volume, (B) cortical tissue volume and (C) cortical bone fraction in the mdx mice in group 2 who were given 4 weeks of prednisolone compared to group 1. The addition of rhGH and rhIGF-1 in group 3 did not rescue the cortical bone deficit. (D) and (E) show an increase in trabecular number (Tb.N) and corresponding reduction in trabecular separation (Tb.S) in groups 2 and 3 compared to group 1 and (F) an overall increase in trabecular bone fraction (BVTV) in groups 2 and 3 compared to control (group 1). (G) shows representative image of mid diaphyseal cortical bone in (i) group 1 (vehicle only), (ii) group 2 (pred and vehicle) and (iii) group 3 (pred, rhGH and rhIGF-1) (H) shows representative image of metaphyseal trabecular bone in (i) group 1 (vehicle only), (ii) group 2 (pred and vehicle) and (iii) group 3 (pred, rhGH and rhIGF-1). Data are presented as mean (± s.d.). *P  < 0.05, **P  < 0.01, ***P  < 0.001 when compared to group 1. *n*  = 8 in group 1 and 6 each in groups 2 and 3. A full colour version of this figure is available at https://doi.org/10.1530/JOE-21-0388.

**Figure 3:**
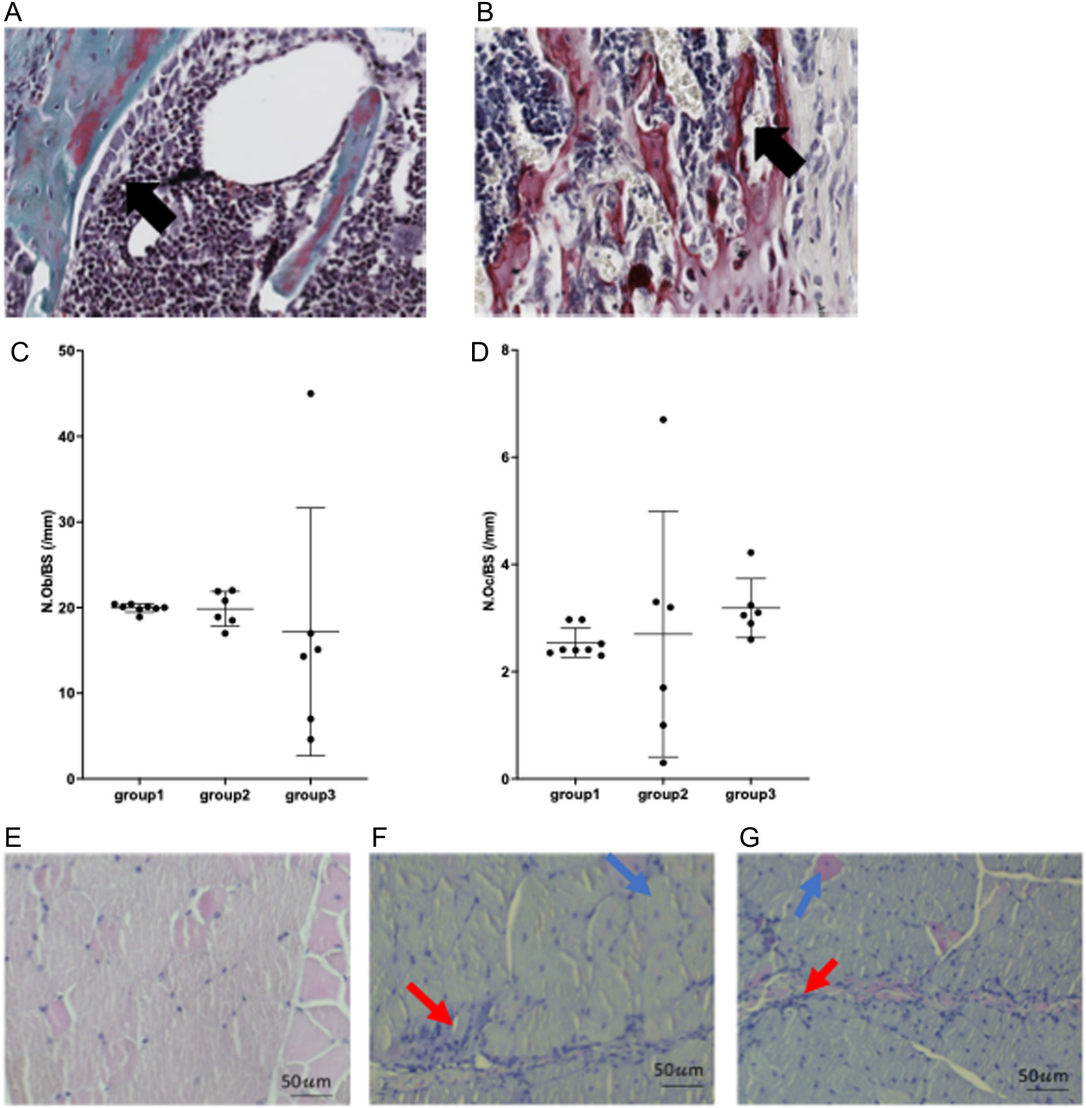
(A) Example of Goldner’s trichome-stained section from proximal tibia metaphysis of a 9-week-old *mdx* mouse from group 1 (after 4 weeks of cherry syrup vehicle) showing cuboidal shaped osteoblasts (arrow) on trabecular bone surface. (B) Representative TRAP activity (arrow) and fast-red stained image of trabecular bone from proximal tibia metaphysis of 9-week-old *mdx* mouse from group 1 (4 weeks of syrup vehicle). (C) Quantification of osteoblast number/bone surface by intervention group in *mdx* mice culled at 9 weeks of age. Data are presented as mean (±s.d.). (D) Quantification of osteoclast number/bone surface by intervention group in *mdx* mice culled at 9 weeks of age. Data are presented as mean (±s.d.). (E) Example of H&E stained section of TA from 9-week-old WT mouse showing normal, regular myofibres with peripheral nuclei (black arrow) and intact sarcoplasm. (F) Example of H&E stained section of TA from 9-week-old *mdx* mouse after 4 weeks of GC: some normal, regular myofibres with peripheral nuclei and intact sarcoplasm, many inflammatory cells (red arrow) and some evidence of regeneration (blue arrow). (G) Example of H&E stained section of TA from 9-week-old mdx mouse after 4 weeks of GC, GH and IGF-1: some normal, regular myofibres with peripheral nuclei and intact sarcoplasm, many inflammatory cells (red arrow) and some evidence of regeneration (blue arrow). A full colour version of this figure is available at https://doi.org/10.1530/JOE-21-0388.

Pred treatment caused a significant reduction in cortical bone parameters and the addition of GH and IGF-1 was not able to rescue this deficit. Cortical bone area (Ct Bar), bone fraction (BV/TV), tissue area (Ct.Tar) and volume (Ct.TV) and bone volume (Ct.BV) were all significantly lower in two intervention groups compared to the control group (Fig. 2 and Table 2). There were no significant differences in tissue mineral density (TMD) by intervention group. Pred treatment caused an increase in trabecular number (Tb.N), bone fraction (BV/TV) and connectivity (Conn) and a reduction in trabecular thickness (Tb.Th) and separation (Tb.S) compared to the control group. The addition of GH and IGF-1 did not alter these findings (Fig. 2 and Table 2). There were no significant differences in the biomechanical properties of the *mdx* tibiae, αCTX or P1NP levels, the percentage of PCNA positive nuclei seen in chondrocytes of the proximal tibial GP, or either osteoclast or osteoblast number/bone surface by intervention group (Fig. 3)

**Table 2:** Trabecular and cortical bone parameters from µCT in *mdx* mice at 9 weeks of age, after either 4 weeks of (1) vehicle only, (2) pred + vehicle or (3) pred + rhGH + rhIGF-1.

	Control	Prednisolone	Prednisolone, rhGH and IGF-1
Trabecular bone parameters			
TV, mm^3^	2.54 (0.21)	2.52 (0.18)	2.69 (0.31)
BV, mm^3^	0.45 (0.08)	0.54 (0.05)	0.55 (0.09)
TV/BV, %	17.60 (2.18)	21.55 (1.30)^a^	20.38 (2.37)^a^
Tb.N, 1/mm	3.63 (0.43)	5.27 (0.52)^b^	5.15 (0.62)^b^
Tb.Th, mm	0.05 (0.002)	0.04 (0.002)^b^	0.04 (0.002)^b^
Tb.S, mm	0.16 (0.01)	0.12 (0.01)^b^	0.13 (0.01)^b^
SMI	1.87 (0.11)	1.80 (0.13)	1.89 (0.14)
Conn	1202 (305)	2460 (545)^b^	2703 (372)^b^
Cortical bone parameters			
TMD g/cm^3^	1.28 (0.12)	1.33 (0.10)	1.30 (0.04)
Ct.TAr,mm^2^	1.08 (0.05)	0.87 (0.05)^b^	0.88 (0.09)^b^
Ps Pm, mm	4.12 (0.44)	3.58 (0.14)^a^	3.60 (0.19)^a^
Ct.Bar, mm^2^	0.71 (0.05)	0.51 (0.03)^b^	0.53 (0.06)^b^
Es.Pm, mm	2.29 (0.07)	2.28 (0.08)	2.28 (0.12)
Ct.TV, mm^3^	0.97 (0.04)	0.78 (0.05)^c^	0.79 (0.08)^c^
Ct.BV, mm^3^	0.64 (0.04)	0.46 (0.03)^b^	0.47 (0.06)^b^
TV/BV, %	66.18 (2.20)	58.82 (1.17)^b^	59.42 (2.68)^c^
J, mm^4^	0.19 (0.06)	0.10 (0.01)	0.11 (0.02)
Ct.Th, mm	0.58 (0.02)	0.57 (0.02)	0.57 (0.03)
Ecc	0.67 (0.08)	0.49 (0.11)^c^	0.54 (0.12)

Data are presented as mean (± s.d.) ^a^*P*  < 0.01, ^b^*P*  < 0.001, ^c^*P*  < 0.05, compared to control group (1). *n*  = 8 in group 1 and 6 each in groups 2 and 3.

BV, bone volume; Conn, connectivity; Ct.Bar, cortical bone area; Ct.BV, cortical bone volume; Ct.Tar, cortical tissue area; Ct.Th, cortical thickness; Ct.TV, cortical tissue volume; Ecc, eccentricity; Es.Pm, endosteal perimeter; J, polar moment of inertia; Ps.Pm, periosteal perimeter; SMI, structural model index; Tb.N, trabecular number; Tb.S, trabecular separation; Tb.Th, trabecular thickness; TMD, tissue mineral density; TV, tissue volume; TVBV, bone fraction.

## Discussion

GC-treated mice showed evidence of growth retardation with a lower BW at cull and lower gains in crown-to-rump and tail length during the study. There was an increase in overall somatic growth rate during of combination rhGH + IGF-1 therapy in the GC-treated *mdx* mice, as demonstrated by increases in both crown-rump length and tail length compared to the GC-treated mice. There was, however, no corresponding increase in tibial length or chondrocytic parameters during the same time period. The reason for this discrepancy between somatic and skeletal growth is not entirely clear but may be related to the differing effects of GC, GH and IGF-1 on trabecular vs cortical bone. Although gain in BW was not significantly different after GH and IGF-1 treatment, BW does not necessarily reflect linear growth (Lupu *et al.* 2001) and is, at best, a crude marker of growth. GH changes body composition and has been shown to reduce the percentage of fat tissue, which may confound any apparent changes in BW gain noted (Kasukawa *et al.* 2003). The effects of GH on BW also appear to be sexually dimorphic, with a lesser effect seen in male mice (Eckstein *et al.* 2004). Consistent with our data, the inhibitory effects of GC on bone growth and body weight were not overcome by GH alone in a recent study of *mdx* mice (Yoon *et al.* 2019*a*).

In addition to the improved somatic growth, this study has shown that pred given either alone or together with rhGH + IGF-1 for 4 weeks improves both absolute and BW-normalised grip strength in juvenile *mdx* mice. Absolute grip strength data also suggested that there was an additional increase in muscle function in the mice given GH + IGF-1, compared to those mice given GC alone. This is consistent with a study where IGF-1 given to *mdx* mice (aged 5–6 weeks of age) for 8 weeks at the same dose as in the current study resulted in a 49% increase in muscle contraction force (Gregorevic *et al.* 2004). Grip strength was used as a surrogate measure of muscle strength. Hindlimb muscle was used as it is readily accessible, and transverse sections are easily obtained and it contains mainly fast myofibres; similar changes with age are seen in the forelimb muscles, which were used to assess grip strength (Grounds 2014*b*). Testes weight was not significantly different between groups, suggesting that pubertal development/sex hormone production was not a confounding factor when assessing strength.

The GC-induced improvements in grip strength concur with the clinical observation that GC stabilise muscle function in boys with DMD (Ricotti *et al.* 2013). Motor function remained unchanged in the recently published clinical study of IGF-1 and in previous studies of GH in boys with DMD (Cittadini *et al.* 2003, Rutter *et al.* 2012, 2020). It is notable that the serum IGF-1 concentrations did not significantly change within the 6 months duration of the clinical study; therefore, it is possible that the predominant actions that were observed in their study may have been via a local rather than systemic effect (Rutter *et al.* 2020).

This study did not demonstrate a significant change in the muscle damage or regeneration parameters measured by histology of the TA in either the GC-treated *mdx* mice or those also given GH + IGF-1 compared to controls. This is in contrast to data from other studies, using the *mdx* mouse, which have suggested that transgenic overexpression of IGF-1 in dystrophic mice led to increased muscle fibre size and number and a reduction in myofibre necrosis (Barton *et al.* 2002, Shavlakadze *et al.* 2004), and other work using GH in the *mdx* mouse that found a change in muscle fibre type and a reduction in regeneration parameters (Yoon *et al.* 2019*a*). The results are, however, in keeping with the study by Gregorevic and colleagues who did not report an increase in myofibre size. In their study, they found a four-fold increase in serum IGF-1 but no change in muscle IGF-1 concentration. This suggests that a muscle-specific-targeted overexpression may be required to directly affect myofibre necrosis and subsequent regeneration (Gregorevic *et al.* 2004). There are no clinical studies that have comparable histological data.

The absence of an increase in bone turnover markers in this study has also been seen in other animal studies; a reduction in bone turnover markers was reported in GH over-expressing transgenic mice (Eckstein *et al.* 2004), and the recently published study of GH in *mdx*mice showed only a minimal effect of GH on bone mineral density (BMD) and microarchitecture (Yoon *et al.* 2019*a*). In the Yoon *et al.* study, there did, however, appear to be a reduction in osteoclast number in GC-treated *mdx* mice who were also given GH. This was thought to be perhaps due to a reduction in the expression of some inflammatory markers; IL-6, IL-1β and FGF21. In humans, IGF-1 at high doses has been shown to increase biochemical markers of bone turnover, but at low doses, the effect seems to be limited to osteoblast function, without a corresponding effect on bone resorption (Grinspoon *et al.* 1996). Serum markers also only provide indirect evidence of bone formation and resorption rates. However, no significant differences were seen in either osteoclast or osteoblast number per bone surface by intervention type, and chondrocyte proliferation rate within the GP remained unchanged between any of the intervention groups. There were also no differences in biomechanical properties assessed by three-point bending. Although both hormones are known to influence osteoblast growth and differentiation (Zhao *et al.* 2000), it is possible that by 9 weeks, the growth rate had slowed sufficiently, such that small differences in osteoblast or osteoclast number, or chondrocyte proliferation rate within the GP in the mice treated with a combination of rhGH and IGF-1, would not be identified. Consistent with this, the addition of IGF-1 and rhGH did not induce a significant increase in tibial bone length. IGF-1 acts as a systemic and local regulator of osteoblast function (Gazzerro & Canalis 2006). Systemic IGF-1 appears to contribute more to cortical bone integrity, while local IGF-1 seems to have a greater role in trabecular bone development (Wu *et al.* 2009). It may be that higher doses of either hormone or both would be required to induce an anabolic bone effect. The dose-response effect of combined GH and IGF-1 intervention may also be more complex due to the concomitant administration of GCs. GCs cause osteoporosis by inhibiting Wnt signalling (Canalis *et al.* 2007) and also decrease IGF-1 transcription in osteoblasts (Delany *et al.* 2001). The GC receptor also binds STAT5 and therefore interferes with downstream GH signalling and acts as a functional GH antagonist (Herrington & Carter-Su 2001). The GC-induced cortical bone defect in this study has also been reported elsewhere (Yoon *et al.* 2016, 2019*a*, *b*). The addition of rhGH+ IGF-1 therapy was unable to reverse this. It may be that the circulating levels of GC were too great, such that the addition of GH and IGF-1 at the levels used within this study was insufficient to provide an anabolic stimulus to bone. It is possible that if we had used a different GC dose or method of administration (pulsed vs daily for example) that we may have seen an effect.

Prior to this study, we performed an extensive literature review which highlighted the extensive range of GC doses and regimens used to try and elicit GIO without proving efficiacy of one over the other (Wood *et al.* 2018). Therefore, preliminary dose-finding studies were carried out to find a suitable dose which concluded that prednisolone given at 20 mg/kg/day was sufficient to induce growth retardation and changes in cortical bone in both the WT and mdx mice over a 28-day period. Prednisolone was given by oral gavage to mimic the route used in children with DMD in the United States.

Unlike in human studies of GIO where a plethora of data exists to show that GCs predominantly affect trabecular bone (Van Staa *et al.* 2002), there is less evidence for a trabecular effect in mice. Indeed, we disclosed that trabecular number and connectivity were increased and trabecular separation decreased in both WT and mdx mice after 4 weeks of 20 mg/kg/day prednisolone which was unchanged by GH/IGF-1 treatment. Previous studies have reported similar anabolic effects of GC on trabecular bone (Yoon *et al.* 2018, 2019*a*), whereas others have reported no effects of GCs on trabecular bone (Abe *et al.* 2016, Bergström *et al.* 2018). Furthermore, an increase in trabecular bone volume and BMD in combination with a reduction in cortical bone content and thickness has also been reported in female C57BL6 mice treated with dexamethasone for 2.5 weeks (Grahnemo *et al.* 2015). Nevertheless, studies using older C57BL6 mice have reported trabecular changes in vertebrae suggesting that GC effects on trabecular bone may be site as well as age-specific (Rauch *et al.* 2010).

It is also possible that the 4-week intervention period was sufficient to demonstrate somatic growth-promoting effects but insufficient to enhance individual longitudinal bone growth or structural change; sensitivity to GH in target tissues is known to be time- and tissue-dependent (Kasukawa *et al.* 2003). There is a critical post-weaning growth spurt in mice, with initiation of GH action at approximately 2 weeks of age. This phase of growth peaks at 25 days and then subsequently declines. Studies have shown that growth retardation in mouse mutants lacking the GH or IGF-1 receptor was most marked during this period (Lupu *et al.* 2001). It is possible, therefore, that if the intervention had started earlier, larger changes and also anabolic effects on skeletal development may also have been observed. It is difficult to balance the most appropriate intervention period to identify changes in somatic growth compared to changes in skeletal development (Wood *et al.* 2018). Steady increases in bone parameters of mice are seen until 6 months of age; therefore, if the intervention period was extended, then changes in bone may have become apparent (Eckstein *et al.* 2004) but growth rates would have been too slow by this age to detect any meaningful change. Future studies could include incremental regimens of rhGH and IGF-1 both separately and in combination and the duration could also be increased. Other techniques such as calcein labelling to quantify bone formation rate could also be beneficial.

In summary, for as long as GC remain a part of the routine management of DMD, finding an appropriate anabolic agent for the treatment of GC-induced osteoporosis and GC-induced growth retardation will remain a clinical priority. The current study has shown that the combination of exogenous GH and IGF-1 can increase somatic growth but may not improve the negative effects of long-term GC on longitudinal bone growth or cortical bone development in juvenile *mdx* mice.

## Declaration of interest

Colin Farquharson is a co-editor in chief of the *Journal of Endocrinology*. Colin Farquharson was not involved in the review or editorial process for this paper, on which he is listed as an author. The other authors have nothing to disclose.

## Funding

C W is funded by the Medical Research Council/MDUK (MR/N020588/1). C F is grateful to the Biotechnology and Biological Sciences Research Council (BBSRC) for Institute Strategic Programme Grant Funding BB/P0137321.
